# Supplementary material for: Association of urban forest landscape characteristics with biomass and soil carbon stocks in Harbin City, Northeastern China
Source: PeerJ. 2018 Oct 29;6:e5825. doi: 10.7717/peerj.5825 (PMC6211268; doi:10.7717/peerj.5825)
Supplement: Table S1 [file peerj-06-5825-s002.docx]

**Appendix Table 1** Landscape metrics used in this study and their equations and ecological descriptions

| Index | Equation | Description |
| --- | --- | --- |
| TA | $\frac{1}{10000}\sum a_{i}$ (ha) | Total area. a_i_, area of the ith patch (m^2^). |
| NP | $n_{i}$ | Number of patches. n_i_, number of patches of the focal type in landscape. |
| LPI | $100\frac{\max\left( a_{i} \right)}{A_{T}}$（%） | Largest patch index, percentage of the largest patch of the focal type in the landscape, mainly reflecting configuration. A_T_, total landscape area (m^2^). |
| AREA_MN | $\frac{1}{10000}\frac{\sum a_{i}}{n_{i}}$ (ha) | Mean patch area, mainly reflecting configuration. |
| PARA_AM | $\frac{p_{i}}{a_{i}}$ | Area-weighted Perimeter-Area Ratio, mainly reflecting the complexity of patch shape, ratio of the patch perimeter (m) to area (m^2^). p_ij_, perimeter (m) of patch i. |
| LSI | $\frac{25\sum_{k = 1}^{m} e_{ik}}{\sqrt{A_{T}}}$ | Landscape shape index, mainly reflecting the aggregation/dispersion of patches, e_ik_, total length (m) of edge in landscape between patch types i and k. |
| ENN_MN | $h_{ij}$ (m) | Euclidean Nearest-Neighbor Distance, mainly reflecting the connectivity of patches, h_ij_, distance (m) from patch ij to nearest neighboring patch of the same type |
