# Supplementary material for: Association of urban forest landscape characteristics with biomass and soil carbon stocks in Harbin City, Northeastern China
Source: PeerJ. 2018 Oct 29;6:e5825. doi: 10.7717/peerj.5825 (PMC6211268; doi:10.7717/peerj.5825)
Supplement: Table S2 — Note: D means DBH (cm), H means height (m), CA means crown area (m2), Bag, Br, Bbranch, Bstem, Bleaf means aboveground biomass (kg), root biomass (kg), branch biomass (kg), stem biomass (kg), and leaf biomass (kg), respectively. [file peerj-06-5825-s003.docx]

**Appendix Table 2.** Allometric growth equations used in this paper

| **Latin Name** | **Tree biomass Equations** | **Citations** |
| --- | --- | --- |
| Pinus tabuliformis | B_ag_=B_stem_+B_branch_+B_leaf_; B_stem_=0.11*D^2.34^;  B_branch_=0.01*D^2.58^; B_leaf_=0.0049*D^2.48^; B_r_=0.64*D^2.1^ | Ma(1989); Liu and Li (2012) |
| Ulmus | B_ag_=B_stem_+B_branch_+B_leaf_; B_stem_=0.043*D^2.87^;  B_branch_=0.0074*D^2.67^; B_leaf_=0.0028*D^2.50^ | Chen and Guo (1984); Liu and Li (2012) |
| Picea | B_ag_=B_stem_+B_branch_+B_leaf_; B_stem_=0.057*D^2.48^;  B_branch_=0.012*D^2.41^; B_leaf_=0.083*D^2.37^; B_r_=0.0088*D^2.54^ | Chen and Guo (1984); Liu and Li (2012) |
| Betula platyphylla | B_ag_=102.159*D^2.367^/1000; B_r_=101.358*D^2.518^/1000 | Wang (2006) |
| Populus | B_ag_=101.826*D^2.558^/1000; B_r_=101.025*D^2.56^/1000 | Wang (2006) |
| Pinus koraiensis | B_ag_=102.236*D^2.144^/1000; B_r_=101.296*D^2.376^/1000 | Wang (2006) |
| Larix gmelinii | B_ag_=101.977*D^2.451^/1000; B_r_=101.085*D^2.57^/1000 | Wang (2006) |
| Acer | B_ag_=101.930*D^2.535^/1000; B_r_=102.112*D^1.981^/1000 | Wang (2006) |
| Fraxinus | B_ag_=102.136*D^2.408^/1000; B_r_=101.396*D^2.467^/1000 | Wang (2006) |
| Juglans mandshurica | B_ag_=102.235*D^2.287^/1000; B_r_=101.226*D^2.397^/1000 | Wang (2006) |
| Phellodendron amurense | B_ag_=101.942*D^2.332^/1000; B_r_=101.024*D^2.617^/1000 | Wang (2006) |
| Tilia | B_ag_=101.606*D^2.668^/1000; B_r_=101.273*D^2.452^/1000 | Wang (2006) |
| Quercus mongolica | B_ag_=102.002*D^2.456^/1000; B_r_=101.482*D^2.356^/1000 | Wang (2006) |
| Pinus sylvestrisL.var.sylvestriformis | B_ag_=Bstem+Bbr+Bleaf; B_r_=200.0322*D^1.495^/1000;  B_stem_=0.0159368*D^2.949^+0.6300862*D^0.759^;  B_branch_=0.0557699*D^2.483^; B_leaf_=0.1090*D^4.293^/1000 | Zou, Pu (1995) |
| Pinus sylvestrisvar.mongolicaLitv | B_ag_=B_stem_+B_branch_+B_leaf_; B_stem_=0.0439*(D^2^H)^0.8852;^  B_branch_=0.02388D^4.1912^H^-2.3076^; B_leaf_=0.1082D^2.7169^H^-1.3955^ | Jia, Jiang (2008) |
| Platycladus | B_ag_=B_stem_+B_branch_+B_leaf_; B_stem_=0.013(D^2^H)^0.5969^+0.0036(D^2^H)^0.6758^; B_branch_=0.00274(D^2^H)^0.5973^+0.004965(D^2^H)^0.5975^+0.00055(D^2^H)^0.5879^; B_leaf_=0.003787(D^2^H)^0.5976^ | Chang, Che (1997) |
| Padusracemosa | B_ag_=0.00009D^2.696^; B_r_=0.035D^2.641^/1000 | Li (2010) |
| Rosaceae | B_ag_=10-0.6657*D^1.7041^ | Wu (2012) |
| Tree generalized equation | B_ag_=101.945*D^2.467^/1000; B_total_=102.033*D^2.469^/1000; B_r_=B_total_-B_ag_ | Wang (2006) |
| Acer ginnala | B_ag_=0.527D^2.217^/1000; B_r_=0.149D^2.261^/1000 | Li (2010) |
| Syringa reticulata | B_ag_=0.395D^2.3^/1000; B_r_=0.129D^2.302^/1000 | Li (2010) |
| Euonymus alatus | B_ag_=0.095D^2.655^/1000; B_r_=0.089D^2.291^/1000 | Li (2010) |
| Rhamnusschneideri | B_ag_=0.169D^2.555^/1000; B_r_=0.092D^2.314^/1000 | Li (2010) |
| Viburnum sargenti | B_ag_=0.141D^2.649^/1000;; B_r_=0.245D^1.994^/1000 | Li (2010) |
| Tree Shrub generalized equation | B_ag_=0.182D^2.487^/1000; B_r_=0.089D^2.37^/1000 | Li (2010) |

Note: D means DBH (cm), H means height (m), CA means crown area (m^2^), B_ag_, B_r_, B_branch_, B_stem_ , B_leaf_ means aboveground biomass (kg), root biomass (kg), branch biomass (kg), stem biomass (kg), and leaf biomass (kg), respectively.

Chang, X.X., Che, K.J., Song, C.F., Li, B.X. 1997. Biomass and Nutrient Element Accumulation of Sabina prez ewalskii Foret Community. Journal of Northwest Forestry College 01: 24-29

Chen, C., Guo,X. 1984. Research on biomass of broad-leaved Korean pine forest. Forest Investigation Design 02: 10-19

Jia, W., Jiang, S., Li, F.R. 2008. Biomass of Single Tree of Pinus sylvestris var.mongolica in Eastern Heilongjiang. Journal of Liaoning Forestry Science &Technology 03: 5-9

Li, X. 2010. Biomass allometry and allocation of common understory in a natural secondary forest in Maoershan, Northeast China. Master of Science, Thesis, Notheast Forestry University

Liu, C., Li, X. 2012. C storage and sequestration by urban forests in Shenyang, China. Urban Forestry & Urban Greening 2: 121-128. DOI: [10.1016/j.ufug.2011.03.002](https://doi.org/10.1016/j.ufug.2011.03.002)

Ma, Y.A. 1989. Study on the bimass of Chinese Pine forests. Journal of Beijing Forestry University 04: 1-10

Wang, C.K. 2006. Biomass allometric equations for 10 co-occurring tree species in Chinese temperate forests. Forest Ecology and Management 222(1-3): 9-16. DOI: [10.1016/j.foreco.2005.10.074](https://doi.org/10.1016/j.foreco.2005.10.074)

Wu, F. 2012. Appraisal of C storage in Urban forest patches and its distribution pattern in Maanshan City, Master of Science, Thesis, Anhui Agricultural University

Zou, C., Pu, J., Xu, W. 1995. Biomass and productivity of Pinus sylvestrif ormis plantation. Chinese Journal of Applied Ecology, 02: 123-127
